# Stimulus-invariant auditory cortex threat encoding during fear conditioning with simple and complex sounds

**DOI:** 10.1016/j.neuroimage.2017.11.009

**Published:** 2018-02-01

**Authors:** Matthias Staib, Dominik R. Bach

**Affiliations:** a Division of Clinical Psychiatry Research, Psychiatric Hospital, 8032, University of Zurich, Switzerland; b Neuroscience Centre Zurich, 8057, University of Zurich, Switzerland; c Department of Psychiatry, Psychotherapy, and Psychsosomatics, Psychiatric Hospital, 8032, University of Zurich, Switzerland; d Wellcome Trust Centre for Neuroimaging, University College London, London WC1 3BG, United Kingdom

**Keywords:** Fear conditioning, Threat prediction, Multivariate pattern analysis (MVPA), Functional MRI (fMRI), Auditory cortex

## Abstract

Learning to predict threat depends on amygdala plasticity and does not require auditory cortex (ACX) when threat predictors (conditioned stimuli, CS) are simple sine tones. However, ACX is required in rodents to learn from some naturally occurring CS. Yet, the precise function of ACX, and whether it differs for different CS types, is unknown. Here, we address how ACX encodes threat predictions during human fear conditioning using functional magnetic resonance imaging (fMRI) with multivariate pattern analysis. As in previous rodent work, CS+ and CS- were defined either by direction of frequency modulation (complex) or by frequency of pure tones (simple). In an instructed non-reinforcement context, different sets of simple and complex sounds were always presented without reinforcement (neutral sounds, NS). Threat encoding was measured by separation of fMRI response patterns induced by CS+/CS-, or similar NS1/NS2 pairs. We found that fMRI patterns in Heschl's gyrus encoded threat prediction over and above encoding the physical stimulus features also present in NS, i.e. CS+/CS- could be separated better than NS1/NS2. This was the case both for simple and complex CS. Furthermore, cross-prediction demonstrated that threat representations were similar for simple and complex CS, and thus unlikely to emerge from stimulus-specific top-down, or learning-induced, receptive field plasticity. Searchlight analysis across the entire ACX demonstrated further threat representations in a region including BA22 and BA42. However, in this region, patterns were distinct for simple and complex sounds, and could thus potentially arise from receptive field plasticity. Strikingly, across participants, individual size of Heschl's gyrus predicted strength of fear learning for complex sounds. Overall, our findings suggest that ACX represents threat predictions, and that Heschl's gyrus contains a threat representation that is invariant across physical stimulus categories.

## Introduction

Learning to predict threat from neutral precursors is crucial for survival in biological environments. Fear conditioning entails establishing an association between such precursors (conditioned stimuli CS), and an aversive event (unconditioned stimulus, US). Non-human animal research has provided compelling evidence that this association is formed within a subcortical fear learning network that encompasses the amygdala as a key structure for storing CS/US associations (Campeau and Davis, 1995, LeDoux, 2003). Although post-learning plastic receptive field changes are observed in sensory cortices (Weinberger, 2007), synaptic plasticity in the amygdala suffices to learn threat predictions from pure sine tones with a single frequency. Indeed, sensory cortex lesions in non-human animals leave fear acquisition intact (Romanski and LeDoux, 1992a). However, for naturally occurring sounds such as frequency-modulated sweeps, which contain multiple frequencies with a temporal pattern, there is evidence in rodents that ACX lesions impair fear learning (Ohl et al., 1999, Peter et al., 2012). This could indicate that sensory cortices, and specifically ACX, are required for some forms of threat conditioning, thus conceptually expanding beyond a role of the amygdala (Herry and Johansen, 2014). The precise function of ACX during learning, however, remains elusive. In rodents, primary ACX (A1) neurons are activated by non-auditory US via the basal forebrain (Peter et al., 2012). There is a suggestion in rodents that during learning from complex sounds, A1 relays this US signal from basal forebrain to amygdala (Letzkus et al., 2011). This information relay appears necessary for learning from complex sounds as its disruption inhibits fear learning in mice (Letzkus et al., 2011). For simple sounds this US relay has not been investigated. Taken together, these findings imply that at least for complex sounds, CS and US information may converge in A1. This allows for a possibility that threat predictions are encoded in A1 over and above threat prediction in the amygdala. The lack of direct connections between A1 and amygdala (McDonald, 1998, Abivardi and Bach, 2017) as well as the impact of higher auditory areas on auditory fear conditioning with complex sounds (Ohl et al., 1999, Peter et al., 2012) suggest that threat predictions are likely to be found in higher ACX subfields as well.

**Fig. 1:**
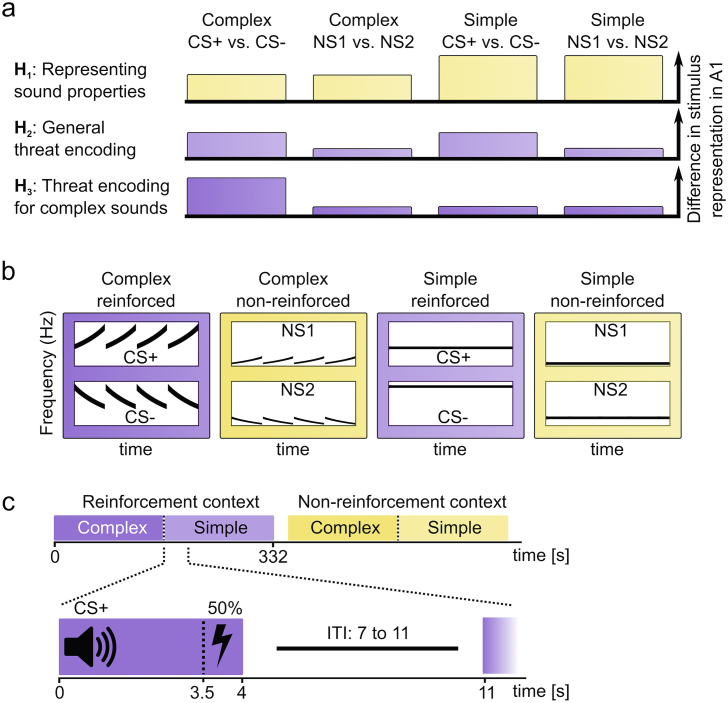
Hypothesis and methods. **(a)** In ACX, we tested three possibilities for CS encoding: (1) CS encoding represents their physical properties and is thus similar to encoding of never-reinforced NS; this may be the same for simple and complex sounds, or different (as illustrated here). (2) CS encoding reflects threat predictions for simple and complex sounds alike, i.e. the difference between CS and NS is the same for simple and complex sounds. (3) CS encoding represents threat only for complex but not simple sounds. An incidental button press task controlled attention and sensory discrimination for all sound pairs. **(b)** Frequency-spectrograms of complex and simple sounds. We compared differential fear conditioning of frequency-modulated sweeps (complex) with single sine tones (simple), **(c)** Fear conditioning paradigm during fMRI. Participants were presented with reinforced and non-reinforced sounds in alternating order. They were instructed about the context (reinforced or non-reinforced) but not about the CS-US associations.

To investigate threat predictions during fear acquisition in humans, we here focus on CS representations in ACX. Such representations have been observed in rodents (Quirk et al., 1995) and humans (Haritha et al., 2013) but it is not clear to what extent they differ between simple or complex CS, and whether they are stimulus-specific. In humans, auditory fear conditioning has only rarely been investigated (see Fullana et al., 2016 for review). Here, we arbitrate between three possible functions of ACX in CS processing (Fig. 1). First, ACX could send a compacted CS identity signal to the amygdala, and separately relay the previously demonstrated US signal, which are then paired within amygdala (hypothesis 1). In this case, the CS response in the ACX would be unaffected by CS/US coupling, i.e. it should merely reflect the physical stimulus properties rather than threat predictions. ACX could also pair CS-US information within the same neural populations and form threat predictions. In this case, CS responses in ACX would depend on whether or not a CS is coupled with US. Such predictions may be represented equally for simple and complex CS (hypothesis 2), or only when learning from complex sounds (hypothesis 3).

To distinguish these possibilities, we used high-resolution functional magnetic resonance imaging (MRI) in humans, together with multivariate pattern analysis (MVPA) to assess differences in information encoding (Kriegeskorte et al., 2006, Norman et al., 2006). Specifically, we analysed to what extent CS+/CS- responses could be distinguished in ACX. Crucially, CS+/CS- do not only differ by their threat association, but also in physical features. Hence, we compared the distinction of CS+/CS- responses from the distinction of similar sound pairs, NS1/NS2, that only differed physically but were never paired with US. In a reinforcement context, indicated by screen background colour, a complex sound (CS+, Fig. 1) composed of four sweeps, co-terminated with an aversive electrical shock in 50% of trials, while CS- was always presented alone. In a neutral control context, two neutral sounds (NS1/NS2) with similar temporal pattern but different principal frequency were always presented alone. To analyse possible differences in threat encoding between complex and simple sounds, we included another set of four simple (pure sine) tones. Fear learning was confirmed by measuring anticipatory sympathetic arousal, and was similar for simple and complex CS. Crucially, we disregard all trials in which a US occurs because we cannot disambiguate BOLD responses to CS and US in our fMRI approach.

## Materials & methods

### Participants

Eighteen healthy volunteers (ten female, mean age: 24.2 years, age range 18–34) participated in the fMRI experiment and received monetary compensation. All participants were right-handed with normal or corrected to normal vision, had no structural brain abnormalities, and no neurological or psychiatric history. Twenty different volunteers (11 female, mean age: 23.3, age range 18–28) participated in a control experiment outside the MR scanner, in which we sought to confirm in more noise-free conditions that sound type had no impact on behavioural fear learning. No participants were excluded. All participants gave written informed consent. The experiment, including the form of taking consent, was conducted in accordance with the Declaration of Helsinki and was approved by the governmental ethics committee (KEK-ZH 2013-0258).

### Design

The MRI experiment followed a repeated-measures 3-way factorial design with the factors stimulus, context, and complexity. In a reinforcement context, participants were repeatedly presented with either two simple or two complex stimuli (CS+, CS-). 50% of the CS+ co-terminated with an unpleasant electric stimulation to the right wrist. CS- were always presented alone. Participants were not instructed about CS+/CS- contingencies with US. Additional simple and complex stimuli (neutral stimuli, NS1/NS2) were presented in different blocks (non-reinforcement context); participants were explicitly instructed that stimuli in these blocks were never reinforced. In total, eight different sounds were used. The behavioural control experiment used the reinforcement context only with 4 stimuli. For fMRI analysis, classification of stimulus pairs per complexity/context combination was analysed, such that our group-level statistical model used a 2 (context) x 2 (complexity) factorial model on permutation-baseline corrected decoding performance.

### Stimuli

All sounds were monophone sine waves of 4 s duration. Simple stimulus pairs had time-invariant frequencies of 100/200 Hz, or 400/800 Hz. Complex sounds were composed of four 1 s repetitions of either rising or falling frequency, from 100 to 200 Hz or 400–800 Hz, respectively. Balanced across participants, CS were in the high octave and NS in the low octave, or the vice versa. Within octaves, sweep direction (complex) and pitch (simple) was balanced across participants. Loudness of the sounds was set between 70 and 80 dB(A), and adapted for each sound according to the equal-loudness contour by Fletcher-Munson combined with an expert rating to match perceived loudness between sounds. All stimuli were created in Matlab and converted to sound files with the inbuilt wavwrite function. They were played with Cogent 2000 (Version 2000v1.25; www.vislab.ucl.ac.uk/Cogent) and delivered binaurally using high-fidelity MR-compatible headphones (OPTIME 1, MR Confon, Germany), or HD 518 headsets (Sennheiser, Wendemark-Wennebostel, Germany) respectively in the control experiment.

US were unpleasant electric stimulations consisting of a 500 ms duration, 5 Hz train of square pulses with 200 μs width, delivered via a pin-cathode/ring-anode configuration attached to the dominant forearm. Before the experiment, US intensity was set to a clearly discomforting level by adapting current amplitudes. First, electric current was increased from an undetectable intensity until the participant reported that stimulation reached the pain threshold. Next, 14 shocks with a randomly set intensity below the pain threshold were applied while the subject rated discomfort on a 0% (no shock detected) to 100% (painful) scale. Finally, the stimulation was set just below the pain threshold (mean ± SD: 8.25 ± 2.90 mA). After the experiment, participants re-evaluated shock intensity for the same random shocks as before the experiment. In both experiments, participants reported reduced intensity of the US during re-evaluation (fMRI experiment: *t*_*17*_ = *4.73, p* < *0*.*001*, control experiment: *t*_*19*_ = *4.69, p* < *0*.*001*).

### Experimental task

After the participant was situated in the MRI scanner, all stimuli were introduced in a training session without reinforcement. This session allowed the subject to familiarize with the button-press task. Each sound was played twice while the sound/button press mapping was displayed on screen. Participants were tasked to press one of two buttons operated with index and middle finger. Specifically, they were instructed to press one finger for both a rising sound and for a high pitched sound, both in the high octave and in the low octave. Conversely, they pressed the other button for falling and for low pitched sounds in both octaves. This mapping was balanced across subjects such that all possible mappings were used equally often. The acquisition phase was structured into mini-blocks; within each mini-block only 1 stimulus pair occurred, with either stimulus in the pair corresponding to a different finger.

### Threat acquisition

In the acquisition phase, CS and NS occurred in different contexts, indicated by background colour (yellow or purple). Background colour/context association was balanced across participants. The fMRI experiment consisted of 8 blocks, each separated into 2 miniblocks of 12 trials in pseudo-randomized order. In each miniblock, a pair of either complex or simple stimuli occurred. Miniblocks with complex and simple sounds of the same context were presented in alternating order (balanced over participants), followed by two miniblocks of the other context. This procedure resulted in overall 24 trials for each of 8 stimuli across the entire experiment. The inter-trial interval (ITI) was drawn from either 7, 9 or 11 s, which resulted in an average duration of 332 s per block. Trial order and timing was optimized for fMRI analysis by maximizing the variance in the contrast CS+/CS- of simulated time series (Ulmer and Jansen, 2013).

Participants were explicitly instructed about different contexts but not about the CS-US coupling. They were informed that US occurrence could depend on the CS identity but not on their response. Their task was to quickly press a key during each sound, according to the previously trained mapping. Wrong button presses or reaction times exceeding 3 s were signalled after CS termination by a change in the fixation cross. All participants were able to correctly identify 90–100% of the stimuli during the experiment. In a generalised linear mixed effects model, accuracy (hit rate) was higher (F_1, 3384_ = 7.88; p = 0.005) for simple (97.67%) than complex sounds (95.98%). There was no impact of stimulus or context, or interaction of any factor. Reaction times are shown in Table 1.

**Table 1:** Reaction time statistics. Note that participants were not incentivised to respond quickly and had 3 s time to make a response.

Estimated marginal means (SD) in ms	CS-	CS+	NS1	NS2
Simple	820 (52)	839 (52)	872 (52)	901 (52)
Complex	1058 (52)	1032 (52)	1087 (52)	1171 (52)

Linear mixed effects model	df	F	p	

CS	1, 3276	4.23	0.040	
Complexity	1, 3276	320.58	<0.001	
CS x Complexity	1, 3276	<1	n.s.	
Context	1, 3276	29.97	<0.001	
CS x Context	1, 3276	5.21	<0.025	
Complexity x Context	1, 3276	1.09	n. s.	
CS x Complexity x Context	1, 3276	3.83	0.050	

### fMRI data acquisition

Data were recorded in a 3 T (Philips Achieva, Best, The Netherlands) whole body MRI scanner. Anatomical images were acquired using two high-resolution T1-weighted scans, which were averaged off-line (field of view, 255 × 255 × 180 mm; matrix, 336 × 334; 237 sagittal slices with thickness). Functional images during fear acquisition were recorded using a 1.5 mm isotropic resolution, T2*-weighted echo-planar pulse (EPI) sequence (TR, 2.5 s; echo time, 30 ms; flip angle, 85°; in-plane resolution, 216 × 216 mm; matrix, 144 × 144; 30 interleaved slices with thickness 1.5 mm). Susceptibility artefacts in the amygdala were reduced by a slice tilt of 45° and negative phase-encoding polarity (Weiskopf et al., 2006). At the beginning of each experiment, we acquired B0 field maps (TE, 4.1 and 7.1 ms; TR, 698 ms; matrix size, 80 × 80) using 64 slices covering the whole head.

### fMRI analysis

Pre-processing of EPI data was performed using standard procedures in statistical parametric mapping (SPM12; Wellcome Trust Centre for Neuroimaging, London, UK; http://www.fil.ion.ucl.ac.uk/spm/software/spm). Images were corrected for geometric distortions caused by susceptibility-induced field inhomogeneities. A combined approach was used which corrects for both static distortions and changes in these distortions due to head motion (Andersson et al., 2001, Hutton et al., 2002). The static distortions were calculated for each subject from a B0 field map that was processed using the FieldMap toolbox as implemented in SPM12. Using these parameters, echo-planar images were then realigned and unwarped, a procedure that allows the measured static distortions to be included in the estimation of distortion changes associated with head motion. Motion correction parameters were visually checked for sudden movements; no participant moved more than 4 mm into any direction and all participants were retained for further analysis. Slice time correction was performed to correct for differences in acquisition time of individual brain slices (Sladky et al., 2011). Images were then coregistered to the individual's anatomical T1 image using a 12-parameter affine transformation. For mass-univariate analysis, images were transformed to MNI space based on SPM12 segmentation of T1 images (Ashburner and Friston, 2005). Group-space images were smoothed with an 8 mm FWHM Gaussian kernel. In contrast, MVPA was done in unsmoothed native-space images.

After pre-processing, we estimated trial-by-trial BOLD responses to CS and NS. To this end, we used a general linear model (GLM) that contained one regressor per trial, constructed by convolving a 3.5 s boxcar function per event with a canonical hemodynamic response function. This procedure has been shown appropriate to estimate single-trial BOLD responses at the given inter-trial-interval (method LS-A in Mumford et al., 2012). The US was modelled as a separate regressor across all trials. The resulting design matrix also contained a standard 128 s high-pass filter and motion estimates as covariates of no interest. BOLD responses from reinforced trials were not further analysed in line with previous work (Bach et al., 2011), since conditioned and unconditioned response may overlap on these trials, and residual artefacts from increased motion during US presentation may render estimation of responses imprecise.

### Region of interest definition

Our primary analysis focused on Heschl's gyrus (HG) which contains A1 and parts of A2 (Costa et al., 2011). We then expanded our field of view to include the entire ACX, and therefore ran a searchlight analysis within an anatomically defined mask that included the entire superior temporal gyrus, temporal plane, HG and probabilistically defined A1. Anatomical T1 scans were transformed to standard space using the SPM12 segmentation-based non-linear warp to obtain deformation parameters from MNI to native space (Ashburner and Friston, 2005). Using these parameters, region of interest definitions provided in MNI space by the toolbox Automated Anatomic Labeling (AAL) (Tzourio-Mazoyer et al., 2002) were transformed to individual native space. HG and amygdalae comprised our a priori regions of interest (ROI). We additionally did a searchlight analysis within a mask comprising HG, the AAL definition of STG (which includes temporal plane), and a probabilistically defined mask of A1 (Morosan et al., 2001). This mask was created from the probabilistic A1 mask as provided in the SPM Anatomy toolbox (Eickhoff et al., 2005), thresholded at p = 0.31, resulting in a cluster with a spatial extent similar to a morphometric definition of A1 (Artacho-Pérula et al., 2004). ACX was analysed separately for each hemisphere because representation of frequency-modulated sounds has been reported to be lateralised (Warren et al., 2005, Lee et al., 2011). To investigate a relationship of HG size with learning, we used the number of voxels in the warped (native-space) bilateral HG after correcting for overall brain volume, by regressing out the number of voxels of the entire gray matter mask.

### Multivariate image analysis

We used the SPM12 function spm_searchlight to extract BOLD estimates from our regions of interest and passed them to a support vector machine (LibSVM) (Chang and Lin, 2011) for classification within each task condition.

First, BOLD response estimates for each voxel were independently z-scored across all trials to avoid numeric instability in the MVPA. We then used a three-fold cross-validation scheme in which the SVM is trained on two thirds of data (24 stimuli per task condition) and evaluated on the remaining data (12 stimuli per task condition), i.e. every third trial served as test data. In the reinforced context, there were twice as many CS- than CS+, which makes a binomial test unsuitable for assessing above-chance classification. This is why we estimated chance accuracy through permutations by repeating the classification 1000 times with randomly assigned stimulus labels (Bach et al., 2011). Next, the estimated chance performance for each comparison was subtracted from the classification accuracy obtained from using correct labels. This procedure was performed separately for CS trials from complex and simple sounds in the reinforcement context. The same approach was used for the classification of NS1 vs. NS2, separately for complex and simple NS. However, different from the reinforcement context, all trials were usable for fMRI analysis, since no electric stimulation was delivered. To render the classification procedures between contexts comparable in terms of bias and power, we discarded a random subset of 50% samples from one neutral condition before MVPA. Since this process might result in a bias introduced by the selection of discarded NS, we repeated this procedure for each subject 100 times per permutation and averaged the result.

Cross-prediction was performed similarly, by training a classifier on the CS+/CS- distinction for all 36 simple sounds in the reinforcement context, and testing it on CS+/CS- distinction for complex sounds, and vice versa. Because simple and complex CS+ or CS- were matched in terms of the required key press, we also analysed cross-prediction of NS sounds that required the same key press, i.e. predicting left/right-response complex NS from left/right-response simple NS and vice-versa.

Our analysis focused on differences in information content, and pattern similarity in cross-prediction. Difference in information content can be positive or negative. Also, cross-classification performance can theoretically be below zero if two sets of patterns are systematically more dissimilar than expected by chance. This is different from a more common situation in which the existence of information (which can in theory not be negative) is assessed with MVPA. In this situation, the interpretation of standard statistical tests has been challenged (Allefeld et al., 2016), but this is not the case in the present study.

Searchlight analysis (Kriegeskorte et al., 2006) was done for each participant with a 10 mm moving searchlight for CS classification within each condition, using the SPM function spm_searchlight. Voxel-wise results were written into 3D images and analysed on the group level.

### SCR analysis

Skin conductance was recorded as described previously (Bach et al., 2010a, Bach et al., 2010b, Staib et al., 2015) on thenar/hypothenar of the non-dominant hand. In the MRI scanner, we used a Biopac MP150 data acquisition system coupled to a GSR-100C signal amplifier (BIOPAC Systems, Inc. Camino Goleta, CA) at 1000 Hz sampling frequency. For the control experiment outside the MRI, we used an integrated SCR coupler/amplifier (LabLinc V71-23, Coulbourn) and AD converter (DI-149/Windaq, Dataq) at 200 Hz sampling rate. Fear learning was assessed through model-based estimation of anticipatory sympathetic arousal (Bach et al., 2010a, Bach et al., 2010b, Staib et al., 2015) using the Matlab toolbox PsPM 3.0 (http://pspm.sourceforge.net/). For each trial, the most likely cognitive input that caused the observed skin conductance is estimated as the amplitude of a sudomotor nerve burst in a time window of 0–3.5 s after CS onset, using a canonical SCR response function (Bach et al., 2010a, Bach et al., 2010b), and settings optimized to assess fear learning (Staib et al., 2015). For each participant, trial-wise estimates across all conditions were centred on their mean and divided by their standard deviation, and subsequently averaged within conditions. Data sets of two participants were excluded from analysis due to artefacts during SCR measurement.

### Statistical analysis

Statistical analysis was performed in R 3.3.1 (www.r-project.org) using linear mixed effects models in package nlme 3.1–128 (Pinheiro and Bates, 2006). Single-trial estimated aSA were analysed in a 2 (CS) x 2 (complexity) model for the control group and in a 2 (CS) x 2 (context) x 2 (complexity) model for the fMRI group. Condition-wise MVPA results - i.e. permutation-corrected performance of the CS+/CS- classification - were analysed in a 2 (context) x 2 (complexity) x 2 (hemisphere) model for HG and in a 2 (context) x 2 (complexity) model for all other areas. Cross-prediction results were analysed in a 2 (context) x 2 (direction of cross-prediction) model. All models contained random subject intercepts. Post-hoc contrasts of significant findings were conservatively analysed with Wilcoxon tests, thus accounting for a smaller number of data points. Image-based statistical tests were done using SPM group level analysis with family-wise error correction for multiple comparison at a voxel-inclusion threshold of p < 0.001, using a random-field theory based approach as implemented in SPM (Worsley et al., 1992). All results are reported at p < 0.05 corrected within the ROI mask. Notably, for this combination of voxel-inclusion threshold and p-value, this approach has been shown to suitably control the false positive rate (Eklund et al., 2016). For mass-univariate analysis, we examined the planned contrasts (CS+ > CS-) > (NS1 > NS2) [analogous to the main effect of context in MVPA], (CS+ > CS-) > (NS1 > NS2) complex, (CS+ > CS-) > (NS1 > NS2) simple, (CS+ > CS-), (CS+ > CS-) complex, (CS+ > CS-) simple, (NS1 > NS2), (NS1 > NS2) complex, and (NS1 > NS2) simple.

## Results

### Fear acquisition is similar for complex and simple sounds

**Fig. 2:**
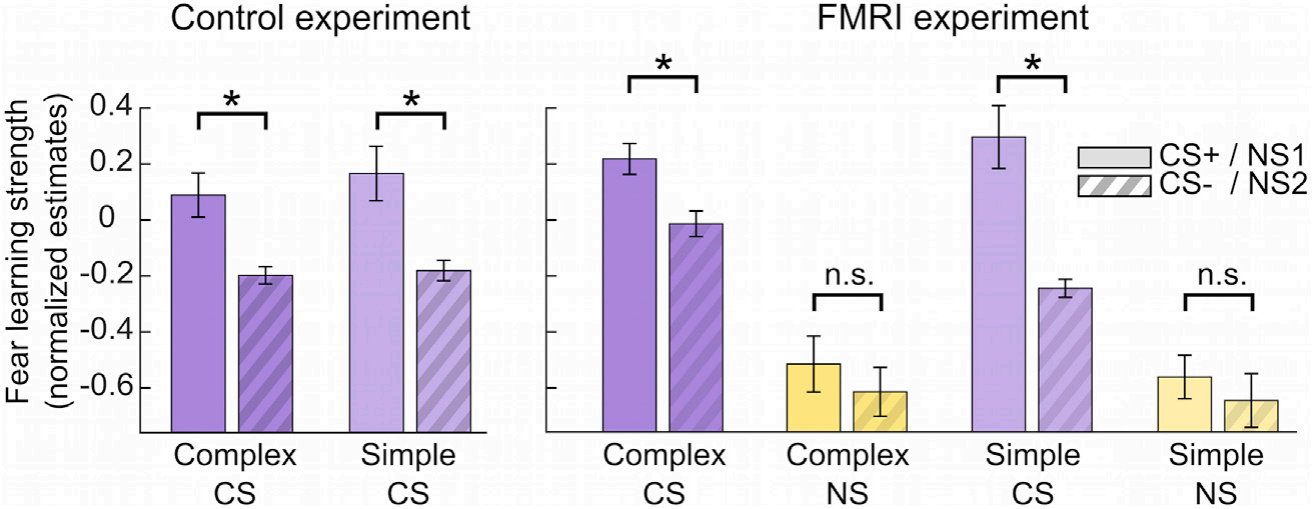
Anticipatory sympathetic arousal estimated from SCR, normalized across trials within each participant (error bars show group-level SEM). Trials where a US was presented are excluded from analysis; **(a)** fear learning outside the MR scanner and **(b)** during fMRI acquisition. Results indicate fear learning for CS (purple) and no differences in arousal for the non-reinforced context (yellow).

We confirmed that participants learned an association between CS and US, by comparing anticipatory sympathetic arousal between CS+, CS-, and NS (Fig. 2, Table 2). In a behavioural experiment outside the MRI, with presumably higher signal-to-noise ratio, participants showed fear learning - i.e. higher CS+ than CS- responses - for simple and complex sounds alike (post-hoc Wilcoxon test, simple: p = 0.008; complex: p = 0.002). For participants in the MRI scanner, we observed fear learning in the reinforcement context (CS × context interaction), but this was modulated by complexity with greater CS+/CS- difference for simple sounds. Nevertheless, post-hoc tests demonstrated fear learning both for simple (p = 0.002) and for complex reinforced sounds (p = 0.044).

**Table 2:** Linear mixed effects model on anticipatory arousal during fear learning.

	Control sample (outside fMRI)			fMRI sample		
	df	F	p	df	F	p
CS	1, 1897	52.3	<0.0001	1, 2665	9.0	0.0028
Complexity	1, 1897	<1	n. s.	1, 2665	6.9	0.0084
CS x Complexity	1, 1897	<1	n. s.	1, 2665	4.2	0.0410
Context				1, 2665	408.6	<0.0001
CS x Context				1, 2665	21.1	<0.0001
Complexity x Context				1, 2665	1.3	n. s.
CS x Complexity x Context				1, 2665	6.0	0.0147

### Threat encoding in HG

Next, we investigated the encoding of threat information in HG. To this end, we analysed for each context and complexity level whether BOLD patterns to CS+/CS-, or pairs of NS, could be distinguished with a cross-validated SVM. Separation of NS quantified the representation of physical stimulus features, and any increase over and above this benchmark in the reinforced context indicates a representation of threat associated with the CS+.

**Fig. 3:**
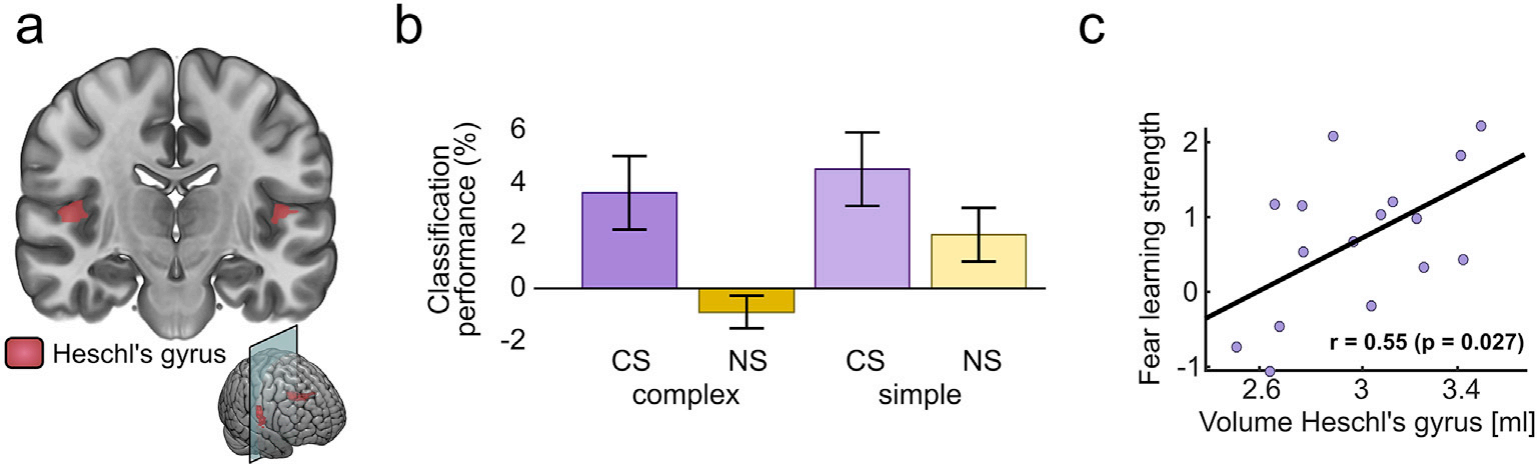
MVPA results. Error bars show standard error of the mean (SEM). **(a)** Regions of interest based on an anatomical atlas were HG and amygdala (not shown). **(b)** In HG, decoding of CS+/CS- was higher than for pairs of NS. Results are collapsed across hemispheres. **(c)** HG size predicts fear learning for complex sounds.

Classification performance is shown here as increase above chance. For all conditions, chance levels of the SVM were between 59 and 61%, determined by 1000 random CS label permutations. Chance levels deviate from 50% due to different number of CS labels. Across all conditions, classification performance above chance was in a similar range as in previous work on fear conditioning (Bach et al., 2011), and decision making (Soon et al., 2013). We compared decoding performances in these four conditions (CS+/CS-, NS1/NS2) in a linear mixed-effects model with the factors context, complexity, and hemisphere (Fig. 3, Table 3).

**Table 3:** Linear mixed effects model on permutation-corrected MVPA performance for classification of CS+/CS−.

Effect	Heschl's gyrus			Amygdala		
	df	F	p	df	F	p
Context	1, 119	21.6	<0.0001	1, 51	15.0	0.0003
Complexity	1, 119	6.4	0.0128	1, 51	5.2	0.0271
Complexity x Context	1, 119	1.8	n. s.	1, 51	6.3	0.0150
Hemisphere	1, 119	<1	n. s.			
Hemisphere x Context	1, 119	<1	n. s.			
Hemisphere x Complexity	1, 119	<1	n. s.			
Hemisphere x Complexity x Context	1, 119	<1	n. s.			

We found a significantly better classification for CS+/CS- than for the physically similar NS1/NS2 (main effect of context) in HG (Fig. 3, Table 3). Classification was better for simple than complex sounds. There was no interaction between these two factors, and no effect involving hemisphere. These findings suggest that threat information of the CS was encoded in HG over and above physical stimulus information also present in NS and both for complex and for simple sounds, thus confirming hypothesis 2. This result prompted us to ask, is threat prediction encoded in a similar way for similar and complex sounds? To answer this question, we used cross-classification, i.e. we asked whether the identity (CS+/CS-) of simple CS could be predicted from a model trained on complex CS, and vice versa. Because motor response was the same for simple and complex CS+, and for simple and complex CS-, respectively, we contrasted CS cross-classification with cross-classification of simple and complex NS that required the same key press. We found that cross-classification performance was significantly higher for CS than for NS (F_1, 119_ = 7.1, p = 0.0087). For CS, averaged cross-classification performance was 1.9% above chance (one-sided Wilcoxon test: p = 0.034), while it was 1.1% below chance for NS (n.s.). These findings suggest that threat encoding had a similar pattern for simple and complex CS+.

**Fig. 4:**
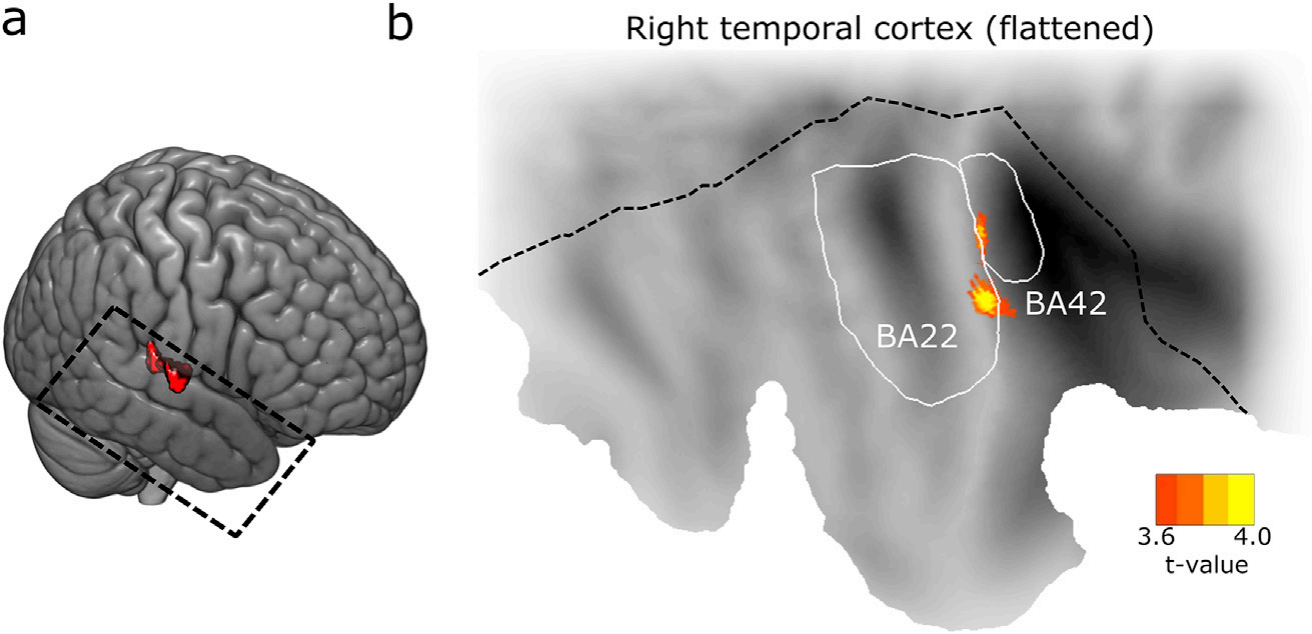
Searchlight results in entire ACX, encompassing HG, STG including temporal plane, and probabilistically defined A1. (a) Significant cluster of classification accuracy CS > NS (red). (b) View on flattened right temporal cortex. The cluster extends from Brodmann area (BA) 42 to BA 22.

Up to here we constrained ourselves to analysing HG. To investigate threat representations within the entire ACX, we performed a searchlight analysis with a moving 10 mm radius searchlight, and analysed the t-contrast CS > NS. This revealed a cluster in ACX, for which CS classification was stronger than NS classification (Fig. 4, peak t = 4.52, peak coordinates 65, -8, 8 in MNI space, volume 1.499 cm^3^). The cluster stretched from Brodmann area 22 (superior temporal area) to 42 (posterior transverse temporal area) of the right hemisphere and included around 10% of HG voxels. Post-hoc analysis of this cluster revealed a significantly better decoding for simple than complex sounds (F_1, 51_ = 13.72, p < 0.001) but no complexity × context interaction (F_1, 51_ < 1), just as in HG (i.e. threat encoding was not specific for simple or complex sounds). However, different from our results in HG, cross-classification performance was similar for CS and NS, i. e. the specific fMRI pattern distinguishing CS ± over and above NS1/2 was not similar between simple and complex sounds. Notably, this searchlight analysis revealed no cluster for the interaction context x complexity, and provided thus no evidence for hypothesis 3.

To explore a causal relationship between ACX anatomy and fear learning, we analysed the relation between HG volume and CS+/CS- difference in aSA. Strikingly, estimated anatomical size of bilateral HG predicted strength of fear learning for complex sounds, even when controlling for total brain volume in the regression (r = 0.55, p = 0.027; Fig. 3c). Such association was not observed for simple sounds, or for amygdala volume.

### Threat encoding in amygdala

The amygdala is crucial for storing threat associations with simple sounds, and also for eliciting fear responses across various CS types. Unsurprisingly, we found threat encoding - i.e. greater CS than NS separation - across both sound types (Table 2). However, this main effect was modulated by complexity (interaction CS x complexity, Table 2): threat encoding was stronger for complex (*p* = .*001*) than for simple (*p* = *0.20*) stimulus pairs. This difference was mainly driven by asymmetric NS encoding: simple NS were better separated than complex NS (*p* = *0.002*) while simple and complex CS were similarly decoded (*p* = *0.90*). Finally, cross-classification demonstrated that simple and complex CS representations were more similar than simple and complex NS representations, suggesting to some degree a similar threat encoding across sound types, as in HG (F_1, 51_ = 4.4, p = 0.0410).

### Mass-univariate results

To exclude that our MVPA results were dominated by more widespread directional differences in BOLD activity between conditions, we performed a mass-univariate analysis. None of the planned contrasts showed a significant result, when correcting for multiple comparison either within the ACX ROI, or within the entire brain.

## Discussion

In this study, we address the functional significance of ACX for fear learning from simple and complex CS, using fMRI in humans combined with MVPA. First, we demonstrate that HG encodes a threat prediction during CS presentation over and above encoding physical properties of the CS, both for simple and for complex auditory stimuli. Using cross-classification, we then show that threat encoding is significantly similar between simple and complex CS. HG includes A1 but also parts of secondary ACX. Next, searchlight analysis within the entire ACX reveals an extended region that also encodes a threat prediction. In this region, however, we found no evidence for similarity of threat encoding associated with simple and complex sounds.

Our result that human ACX encodes a threat prediction from CS extends a previous suggestion that A1 relays US information to amygdala when learning from complex sounds (Letzkus et al., 2011). We suggest that ACX additionally encodes threat prediction from CS before a US occurs. However, although ACX is not required to form threat predictions from simple sounds in rodents, this threat encoding occurs for simple as well as complex sounds - to a comparable extent and in HG also with a similar pattern. Interestingly, we found threat representations in an HG region of interest, but searchlight analysis within ACX including HG revealed a threat-encoding cluster that only encompassed 10% of HG voxels. This may suggest that either threat representations within HG are rather circumscribed, or that the localisation of threat representations within HG is heterogeneous across participants and thus does not impact on searchlight analysis. Such variability could arise either from functional heterogeneity, or from to anatomical variability of auditory areas within HG. HG mainly consists of A1 (core) and A2 (belt) areas (Galaburda and Sanides, 1980). In the present study, it was not possible to distinguish representations these subregions, which is generally difficult based on anatomical information alone (Brugge et al., 2008, Moerel et al., 2012, Wasserthal et al., 2014, Brewer and Barton, 2016). Further fMRI studies using additional functional information, or ECoG recordings, may help resolve the question where precisely these threat representations are localised in terms of functional ACX subfields.

Our multivariate fMRI approach can reveal differential information encoding in neural populations at subvoxel resolution (Kriegeskorte et al., 2006, Norman et al., 2006). It is more challenging to precisely identify the neural populations with fMRI that carry this information. One possibility is that our results relate to early changes in excitatory/inhibitory dysbalance which are induced by US signals received from Nucleus basalis (Froemke et al., 2007) and ultimately - within hours - lead to post-learning receptive field plasticity in A1 (Schreiner and Polley, 2014). Such plasticity has been demonstrated for simple sounds in rodents (Bakin and Weinberger, 1990, Edeline and Weinberger, 1993, Suga and Ma, 2003, Ji and Suga, 2007), and humans (Thiel et al., 2002a, Thiel et al., 2002b, Brockelmann et al., 2011, Kluge et al., 2011), but is not required for fear learning in rodents (Romanski and LeDoux, 1992b). However, our results are unlikely to directly reflect this receptive field retuning. First, receptive field plasticity does not commence before 20 min after start of fear learning and is only complete after some hours (Froemke et al., 2007, Schreiner and Polley, 2014). Secondly, it only occurs for neurons tuned to CS that are paired with the US - and these are different for simple and complex CS in our study. However, we show by cross-classification that threat encoding for simple and complex sounds in HG overlaps. This is difficult to explain with receptive field retuning. We would tentatively suggest that our results in HG reflect a more global CS+ induced response early during learning, induced by the local convergence of CS and US representations. We note that the early stages of CS responses during conditioning are only incompletely understood in non-human species (Schreiner and Polley, 2014).

An alternative explanation for our findings is that CS+ detection by amygdala induces resource prioritisation (e. g. Bach et al., 2015), including selective attention to CS+ in sensory areas. This raises the question whether a CS+/CS- differentiation can be explained by top-down selective attention alone. Crucially, selective attention is thought to alter spectrotemporal receptive fields in A1 to improve detection of the attended stimulus and thus induces changes similar to post-learning receptive field plasticity (Fritz et al., 2010). Indeed, human fMRI studies have shown that the attended stimulus can be decoded from patterns of BOLD signals in auditory areas which implies that these patterns are very specific to the attended-to stimulus (Riecke et al., 2017). In contrast, here we find that not only are simple and complex CS+ responses unspecific, cross-classification shows they can even be significantly decoded from one another. This makes it unlikely that top-down attention accounts for the increased CS+/CS- differentiation in HG.

Taken together, this suggests that our results in HG cannot be explained by post-consolidation receptive field remapping (behaviourally relating to learning-induced selective attention) or top-down selective attention. Furthermore, mass-univariate results show no evidence for global differences in ACX activation as could for example be caused by differential global attention. Thus, our MVPA findings are more likely to stem from a threat encoding mechanism, possibly based on local CS/US convergence. Threat encoding in higher ACX was dissimilar for simple and complex CS and could thus potentially reflect receptive field plasticity although we note that such mechanisms are more thoroughly understood in A1 than other ACX areas (Schreiner and Polley, 2014), and an interpretation of our searchlight results thus remains speculative.

Interestingly, we also observed that estimated HG size predicted behavioural learning indices for complex sounds, lending credence to a causal role of HG in threat learning from complex sounds. Notably, we inferred HG volume from cortex normalisation rather than volumetrically measure HG. Hence, this finding should be replicated in a volumetric or voxel-based morphometeric approach, and possibly in a larger sample.

Our results in the amygdala confirm and extend previous human findings. Synaptic plasticity in amygdala is crucial for fear learning (Clugnet and LeDoux, 1990, Quirk et al., 1995, Blair, 2001). While rodent electrophysiology provides clear evidence that CS+ and CS- responses differ, human fMRI studies have often not reported such differences, as apparent in large meta-analyses (Sehlmeyer et al., 2009, Mechias et al., 2010, Fullana et al., 2016), possibly due to the sparse and interleaved arrangement of neurons responding to CS+ and CS- (Reijmers et al., 2007, Tovote et al., 2015). Multivariate fMRI studies from different laboratories and with two different approaches have provided evidence that CS+ and CS- are encoded differently (Bach et al., 2011, Visser et al., 2011) and that such threat encoding increases over time (Bach et al., 2011). In line with these findings, we observed that both complex and simple CS+/CS- were represented by distinct patterns. Interestingly, this pattern difference was to some extent shared by simple and complex sounds. Complex neutral sounds were not differentially encoded in the amygdala while simple neutral sound pairs showed distinct patterns as well. This lead to a significant CS decoding effect over and above NS only for complex sounds. However, threat encoding is a well-established phenomenon in the amygdala. The non-significant difference between simple CS and simple NS may thus imply that classification performance stemming from encoding of stimulus features (in NS) and from threat encoding (additionally present in CS) is subadditive in the amygdala. Such subadditivity could also apply to HG where the difference between CS and NS was at least descriptively dominated by decoding differences for complex sounds.

Notably, neither amygdala nor HG appears to distinguish neutral complex sounds; yet can apparently encode threat predictions. Thus, it appears that the common representation of CS+/CS- in these areas across complexities is independent from the acoustic features of the sounds and instead is associated with propagation of threat-information to the extended fear-learning network; the ensuing associations may well be created in higher auditory or polymodal regions. Stimulus-independent threat predictions are in line with our initial hypothesis 2 and constrain possible models of amygdala/ACX interactions. The current study could not provide insights into the differential roles of amygdala and ACX in forming threat associations. The relatively low number of trials per condition precluded analysing the trajectory of threat predictions in these areas. More specifically tuned experimental designs might shed light on this question, and electrophysiological methods could help elucidate the intra-trial communication of threat predictions across areas.

In the current study, we focused on threat predictions; however, we cannot disentangle whether our findings are specific to this situation or would also occur for other salient stimuli, or even associative learning of non-salient events. Previous work has highlighted early primary sensory cortex responses to reward predictors (Bach et al., 2017), and these representations have not directly been compared to threat predictors.

Since we excluded all US trials from our analysis, and given the low time resolution of fMRI, we cannot exclude that CS offset responses contribute to our findings. However, a differential offset response to CS- and CS+ must be the consequence of threat predictions such that even in this case, our results highlight ACX threat predictions.

## Conclusion

In summary, we demonstrate a novel pattern of CS-induced threat encoding in HG and higher ACX. HG encoding is similar for simple and complex sounds, making an origin in top-down or post-learning selective (stimulus-specific) attention less likely. Rodent research has suggested that a direct path from thalamus to amygdala, bypassing ACX, is sufficient for acquiring a threat association if sounds are composed of single sine tones, but that A1 is required for complex sounds. Our results indicate that in both cases, threat information from CS is encoded in HG and higher ACX. Our findings strengthen a network perspective on fear acquisition including sensory cortices in humans, encouraging the use of multivariate methods to discover the role of key brain areas in learning environments and shed new light on early processing stages associated with memory formation.

## Funding

This work was funded by the Swiss National Science Foundation [320030_149586/1]. The Wellcome Trust Centre for Neuroimaging is supported by a core grant from the Wellcome Trust [091593/Z/10/Z].
